# Hypolipidemic effects of chitosan and its derivatives in hyperlipidemic rats induced by a high-fat diet

**DOI:** 10.3402/fnr.v60.31137

**Published:** 2016-05-03

**Authors:** Haitao Pan, Qingyun Yang, Guidong Huang, Chen Ding, Peiqiu Cao, Lanlan Huang, Tiancun Xiao, Jiao Guo, Zhengquan Su

**Affiliations:** 1 Key Research Center of Liver Regulation for Hyperlipidemia SATCM/Class III Laboratory of Metabolism SATCM, Guangdong TCM Key Laboratory for Metabolic Diseases, Guangdong Pharmaceutical University, Guangzhou, China; 2 Inorganic Chemistry Laboratory, Oxford University, Oxford, United Kingdom; 3 Guangzhou Boxabio Ltd, D-106 Guangzhou International Business Incubator, Guangzhou Science City, Guangzhou, China

**Keywords:** chitosan, chitosan oligosaccharides, hypolipidemic effect, high-fat diet, PPARα

## Abstract

**Background:**

Hyperlipidemia (HLP) is the primary risk factor of cardiovascular disease (CVD). Various factors, including genetics, physical inactivity, and daily nutritional habits, affect the prevalence of HLP. Recently, it was revealed that dietary fibers, such as pectin, psyllium, and especially chitosan (CTS), may play important roles in hypolipidemic management. Thus, this study aims to determine the hypolipidemic effect and mechanism of CTS and its water-soluble derivatives, chitosan oligosaccharides (M_N_≤1,000 Da (COSI) and M_N_≤3,000 Da (COSIII)), in male hyperlipidemic rats induced by a high-fat diet (HFD).

**Design:**

After the model creation, 120 Sprague-Dawley (SD) rats were equally assigned to 12 groups fed various diets as follows: the normal group with basic diet, an HFD group, an HFD group supplemented with three doses of CTS, COSI and COSIII groups, and an HFD group treated with simvastatin (7 mg/kg·d). After 6 weeks, body weight, fat/body ratio, and the relevant biomarkers of serum, liver, and feces were measured. Additionally, the histological analysis of liver and adipose tissue was performed, and the mRNA expressions of liver peroxisome proliferator-activated receptor-α (PPARα) and hepatic lipase (HL) were examined.

**Results:**

Compared with HFD group, rats fed CTS, COSI, and COSIII showed a better ability to regulate their body weight, liver and cardiac indices, fat/body ratio, as well as serum, liver, and fecal lipids, and simultaneously to maintain the appropriate activity of liver and serum superoxide dismutase (SOD), alanine aminotransferase (ALT), aspartate aminotransferase (AST), as well as liver and fecal total bile acids (TBA). Simultaneously, there had been a higher mRNA expression of PPARα and HL in the treatment groups.

**Conclusion:**

The obtained results suggested that these three function foods can effectively improve liver lipid metabolism by normalizing the expressions of PPARα and HL, and protect liver from the oxidized trauma by enhancing hepatic function, which could be potentially used to remedy hyperlipidemia.

Hyperlipidemia (HLP), characterized by abnormal levels of serum lipids and the dysregulation of lipid metabolism, is the primary risk factor contributing to the formation and progression of atherosclerosis (AS) and subsequent cardiovascular disease (CVD) (1, 2). Recently, increasing studies have focused on how to improve serum lipid concentrations and the absorption of fat in the gastrointestinal tract to remit diet-related chronic diseases, some of which indicated that dietary fibers, such as pectin, psyllium, and chitosan, may display potent hypolipidemic effects (3, 4).

Chitosan (CTS), a kind of de-acetylated derivative of chitin, is a natural polymer of glucosamine derived from the cell walls of some fungi and the exoskeleton of crustaceans, including shrimps, crabs, lobsters, and prawns (5, 6). CTS has beneficial effects on serum lipids, including total cholesterol (TC), triglyceride (TG), and low-density lipoprotein cholesterol (LDL-C), as well as body weight gain (7–9). As the small molecular derivatives of CTS, chitosan oligosaccharides (COS) have shown the better water-solubility and thus can be digested and absorbed more easily by the gastrointestinal tract in animals and humans, which may make them more efficient hypolipidemic and weight-lowering functional food especially in western countries (10–12). Notably in Japan, as the dietary supplements, CTS and COS are the only function foods can be disseminated in public. In this study, we compared the hypolipidemic and hepatoprotective functions of CTS and its derivatives (COSI and COSIII) for the very first time. Additionally, we testified that whether CTS and COS can effectively better the process that liver cholesterol are translated into bile acid, which is useful for improving hyperlipidemia.

To further explore the in-depth molecular mechanisms of CTS and COS regulating the process above, we examined the mRNA expressions of two important regulating factors in the liver: peroxisome proliferator-activated receptor-α (PPARα), a ligand-activated transcription factor involved in the regulation of various aspects of liver lipid metabolisms (13, 14), and its target regulatory proteins, hepatic lipase (HL), which can catalyze TG hydrolysis in chylomicrons (CM) and VLDL-C for tissue use in the form of free fatty acid (FFA) and monoacylglycerol, and provide high-density lipoprotein (HDL) with apolipoproteins and phospholipids for the reverse cholesterol transport (RCT) process (15, 16). Therefore, we speculated about the regulating function of CTS and its derivatives for lipid metabolism via up-regulating the expressions of liver PPARα and HL mRNA levels, which further contributes to the RCT process.

In the present study, we first testified that CTS, COSI, and COSIII can really improve the hepatic cholesterol metabolism by up-regulating the expression levels of liver PPARα and HL mRNA in male hyperlipidemic SD rats induced by HFD. In addition, we also confirmed that the hepatoprotective effects of these foods can significantly contribute to the improvement of liver lipid metabolism in the hyperlipidemic rats.

## Materials and methods

### Materials and regents

CTS (Lot, 10321A; viscosity, >200 mP/s; degree of deacetylation, 96.2%) was purchased from Shandong Aokang Biotech Ltd. (Shandong, China). COSI (Lot, HL130813G; M_N_≤1,000 g.mol^−1^; degree of deacetylation, 95.6%) and COSIII (Lot, HL130921G; M_N_≤3,000 g.mol^−1^; degree of deacetylation, 91.1%) were obtained from Shandong Laizhou Highly Bio-Products Co., Ltd. (Shandong, China). Simvastatin tablets were supplied from Merck Sharp & Dohme Pharmaceutical Company Limited (Hangzhou, Zhejiang, China). Triacylglycerol (TG), total cholesterol (TC), HDL cholesterol (HDL-C), LDL-C, and total bile acid (TBA) kits were purchased from BioSino Bio-technology and Science Inc. (Beijing, China). Superoxide dismutase (SOD), alanine aminotransferase (ALT), and aspartate aminotransferase (AST) assay kits were purchased from the Nanjing Jiancheng Bioengineering Institute (Nanjing, China). All other reagents and solvents were of analytical grade.

### Animals and diets

One-hundred twenty male SPF Sprague–Dawley (SD) rats (weight, 200±20 g; age, 8 weeks) were used for animal experiments. The rats were supplied by the Guangdong Medical Laboratory Animal Center (GMLAC, Guangzhou, China). All animal experimental protocols were approved by the Institutional Animal Care and Use Committee of Guangdong Pharmaceutical University (Guangzhou, China). The rats were maintained in a SPF room at a temperature of 23–25°C, related humidity of 40–70%, and a differential pressure no less than 10 Pa under a constant day-night rhythm. Rats were given water ad libitum throughout the experiments. All animals were provided standard rodent chow (Guangdong Pharmaceutical University Laboratory Animal Center, Guangzhou, China) for 1 week. Then, the 120 rats were divided into two groups: the normal group (NF, *n*=10) fed randomly with the basic diet and the HFD group (*n*=110) were fed an HFD to obtain the hyperlipidemic model.

The basic diet was composed of 20.0% total crude protein, 4.3% total crude fat, 4.8% total crude fiber, 9.7% moisture, 1.19% calcium, 0.77% phosphorus, Ca^2 +^ /P^5 −^ = 1.55, 6.6% crude ash. The test results showed that total bacterial count (<10 cfu/g), the *Escherichia coli* count (<3.0 MPN/100 g), total molds and yeasts (<10 cfu/g), and salmonella (not detected) all met the stipulated standards. The HFD was composed of 52.6% basic feed, 20.0% sucrose, 15.0% lard, 1.2% cholesterol, 0.2% bile salts, 10% casein, 0.6% calcium hydrophosphate, and 0.4% mountain flour in SPF packaging (provided by GMLAC (No. 20130823)). After 2 weeks, blood samples were collected from the orbital vein using a capillary under ether anesthesia. The blood samples were centrifuged (4°C, 3,500 r·min^−1^, 15 min) to obtain plasma samples to determine serum TC, TG, and LDL-C levels, which were the basis of the desired HLP model.

The 110 model rats were randomly divided into 11 groups for the additional feeding study: (1) HFD group (HF) fed with HFD ad libitum only; (2) HFD group treated with simvastatin (7 mg/kg·d) (SV); (3) HFD supplemented with high (1,000 mg/kg·d), middle (500 mg/kg·d), and low (250 mg/kg·d) dose CTS groups (CTS-H, CTS-M, and CTS-L, respectively); (4) HFD supplemented with high (1,000 mg/kg·d), middle (500 mg/kg·d), and low (250 mg/kg·d) dose COSI groups (COSI-H, COSI-M, and COSI-L, respectively); (5) HFD supplemented with high (1,000 mg/kg·d), middle (500 mg/kg·d), and low (250 mg/kg·d) dose COSIII groups (COSIII-H, COSIII-M, and COSIII-L, respectively). The high, middle, and low doses of CTS, COSI, and COSIII were carried out according to reference (17). Each group consisted of 10 animals. The corresponding foods were administered orally by gavage at a dose of 1 mL/100 g per day at the same time for 6 weeks until the study ended. As the control animals, the rats in the HF and NF groups were simultaneously administered with 1 mL/100 g distilled water by the same way above. All rats, including the NF group were sacrificed at 16 weeks old.

### Experimental design

#### Measurement of food intake and body weight gain

The 24 h food intake of rats were recorded every day, body weight, body length, and abdominal girth were measured every week during the experimental period.

#### Measurement of fecal TC, TG and TBA

Three days before the end of the experiments, 72 h feces were collected from each group and dried to a constant weight for smashing and blending. Then, 3.0 mL of 95% ethyl alcohol was used to dissolve 0.25 g of the resulting powder. This suspension was placed in a water bath for three times for 30 min at 60°C until ethyl alcohol volatilization. Then, the solution was transferred to 10 mL of 60% acetic acid to measure TC, TG, and TBA using the respective commercial assay kits.

#### Determination of serum lipids, ALT and AST

Serum was prepared from blood by centrifugation at 3,000 rpm for 15 min at 4°C, and stored at –70°C until the analysis of the lipid parameters. Plasma TC, TG, HDL-C, and LDL-C levels were measured with commercial assay kits using an Automated Biochemistry Analyzer BC200 (Beijing Precil Instrument Co. Ltd, Beijing, China). The activity of plasma ALT and AST were analyzed with ALT and AST assay kits, respectively, using a Mithras LB 940 Multimode Microplate Reader (Berthold Technologies GmBH & Co. KG, Germany).

#### Measurement of fat/body ratio and visceral indices

Rats were subjected to ether anesthesia, sacrificed, and necropsied after serum preparation. The heart, liver, kidney, perirenal white adipose tissue, and epididymal white adipose tissue were quickly removed and weighed on ice. A picture was taken of the whole liver from each animal, and the total weight of the perirenal and epididymal white adipose tissues were used to determine the fat/body ratio (Fat/body ratio = fat mass/body mass). We also measured the wet weight of the heart, liver, and kidneys for each visceral index. Tissues were immediately stored at −80°C following the biopsy for further analysis.

#### Determination of liver lipids, TBA, and SOD

The liver lipid contents were measured as follows: a piece (approximately 0.1 g) of liver tissue was homogenized in chloroform-methanol (1:1, v/v, 2 mL) and the homogenate was extracted with the extracting solution described above (3 mL) by shaking the tubes horizontally for 24 h. The lipid mixtures were centrifuged at 3,000 rpm for 5 min, and the upper aqueous phase was aspirated to determine the liver lipid content using commercial assay kits.

Rat liver TBA contents were determined using the following methods: 0.5 g of liver tissue was homogenized in sodium chloride solution (0.9%, 2 mL) in an ice-water bath, the resulting homogenate (1 mL) was homogenized three times in acetic acid (60%, 9 mL) and the final homogenate was extracted by shaking the tubes horizontally for 10 min. The mixtures were centrifuged at 4,000 rpm for 10 min, and the upper aqueous phase was used to measure liver TBA contents using commercial assay kits.

Liver SOD activity was measured as follows: approximately 0.1 g of liver tissue was homogenized in a sodium chloride solution (0.9%, 0.9 mL) in an ice-water bath. The homogenate was centrifuged at 2,500 rpm for 10 min, and the upper aqueous phase was diluted 10 times to determine liver SOD activity using commercially available analytical kits and the Mithras LB 940 Multimode Microplate Reader.

### Quantitative RT-PCR analysis

Total RNA was isolated from rat livers using TRIzol reagent (Invitrogen, Inc., USA). Single-stranded cDNA was synthesized from 1 µg of total RNA using the PrimeScript^TM^ RT reagent kit with gDNA Eraser (TaKaRa, Code NO. RR047A, Japan) using the following conditions: 37°C for 15 min, 85°C for 5 s, and storage at 4°C. cDNA products were quantified by real-time RT-PCR using a TaKaRa SYBR Premix EX Taq^TM^ kit (TaKaRa, Code NO. RR420A, Japan) and Bio-Rad IQ5 real-time PCR system and analysis software (Applied Biosystems, Canada). The primer sequences used for PCR were designed and synthesized by Sangon Biotech (Shanghai) Co., Ltd. Glyceraldehyde-3-phosphate Dehydrogenase (GAPDH) was used as internal control (Housekeeping gene). The sequences of the forward and reverse primers used for amplification were as follows:

PPARα: Forward: 5′-GAGGTCCGATTCTTCCACTG-3′

Reverse: 5′-GCATCCCGTCTTTGTTCATC-3′

HL: Forward: 5′-AAATCTCCGTTTCCCTGGTG-3′

Reverse: 5′-GGCGGTCACTTTCATCTTTG-3′

GAPDH: Forward: 5′-ACAGCAACAGGGTGGTGGAC-3′

Reverse: 5′-TTTGAGGGTGCAGCGAACTT-3′.

PCR protocols were performed as follows: 95°C for 30 s (initial denaturation), followed by 39–40 cycles at 95°C for 5 s and 60°C for 30 s. Melt curve analyses were performed with each series to confirm the specificity of the primers and amplified products using the following parameters: heated the amplified products from 65°C to 95°C at 0.5°C steps for 5 s. Relative quantification of mRNA expression was analyzed by the 2^−ΔΔCt^ method.

### Histology of different tissues

The liver, white subcutaneous, and mesenteric adipose tissues were cut into 0.5 cm^3^, washed with saline, and placed in the tissue cassette. The cassettes were marked with a pencil, and then placed into 12% formaldehyde solution to fix for 24 h. Residual fixative was washed away by the distilled water. These tissues were dehydrated by 30, 50, 70, 80, 90, 95, and 100% ethanol, embedded in paraffin (BMJ-III embedding machine, Changzhou Electronic Instrument Factory, Jiangsu, China), and then cut into 5 µm thick sections using a the slicing instrument (Leica RM2235; Leica, Heidelberg, Germany). The tissues were stained with hematoxylin and eosin (H&E) and observed under a microscope with 200 magnifications.

### Statistical analysis

All data are expressed as the mean±SE. Differences between groups were determined by one-way ANOVA using SPSS for Windows, version 19.0 (SPSS Inc., Chicago, IL, USA). Significant differences among means were determined using Student–Newman–Keuls multiple range tests, and a *p*<0.05 was considered statistically significant.

## Results and discussion

### Food intake, body weight gain, fat/body ratio, and visceral indices

**Fig. 1:**
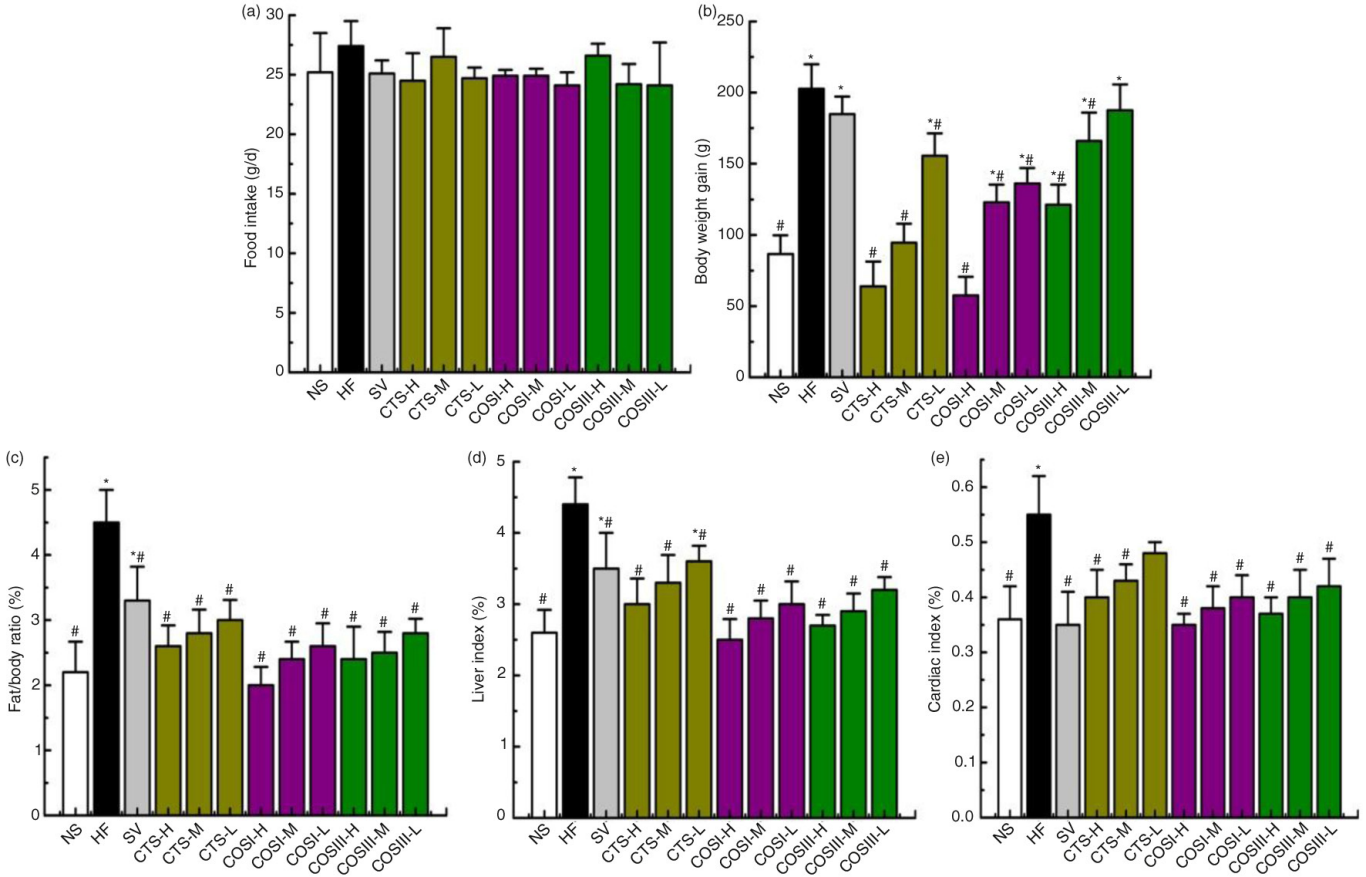
Changes in food intake (a), body weight gain (b), fat/body ratio (c), liver (d), and cardiac (e) indices in hyperlipidemic rats during the treatment (*p<0.05, significant difference when compared with rats in the NF group; #*p*<0.05, significant difference when compared with rats in the HF group; same as Figs. 3, 5, 7, and 10). The data are expressed as means±SD (*n*=10).

**Fig. 2:**
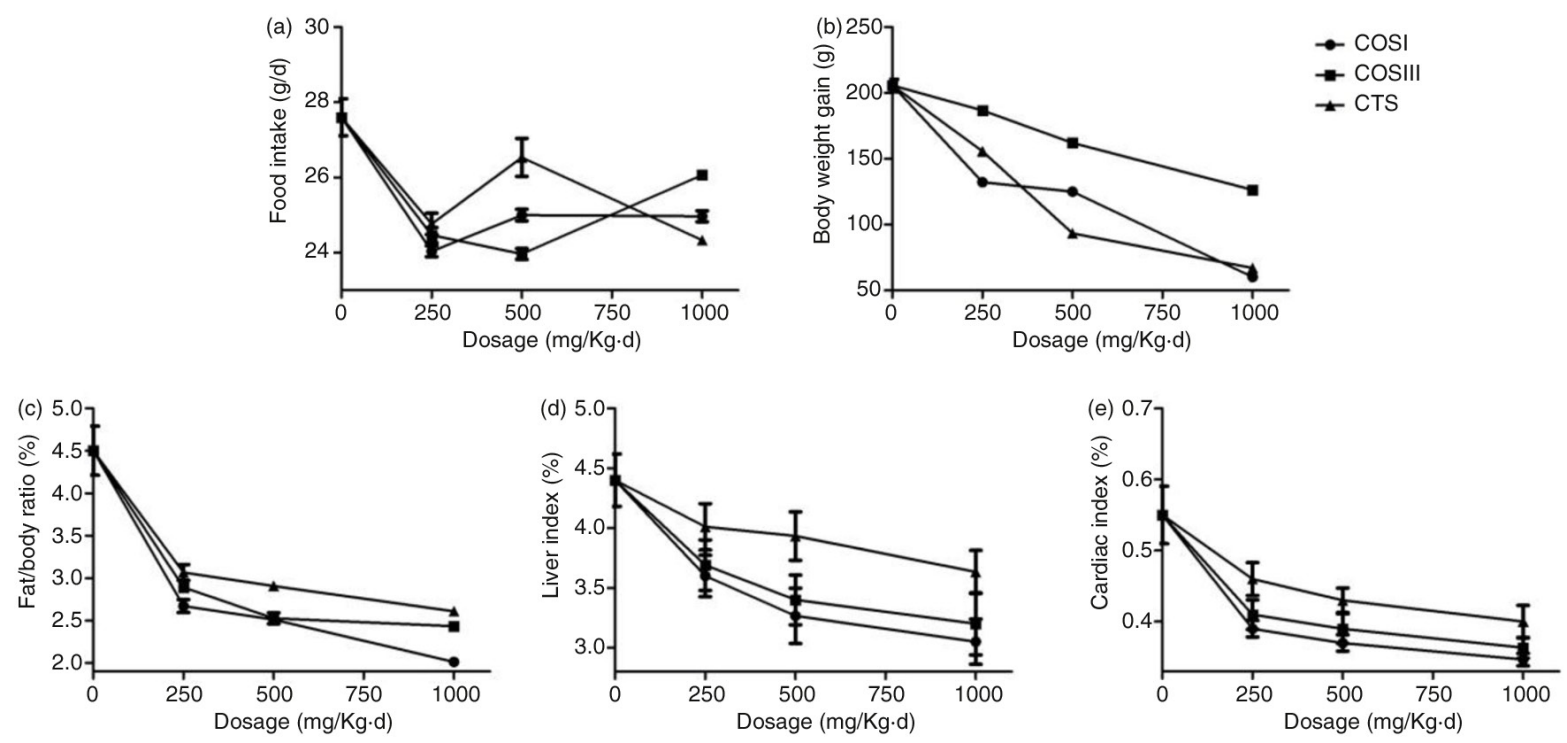
Comparison of different dose COSI, COSIII, and CTS (HFD only, HFD supplemented with low (250 mg/kg·d), middle (500 mg/kg·d), and high (1,000 mg/kg·d) dose COSI, COSIII, and CTS, respectively) in food intake (a), body weight gain (b), fat/body ratio (c), liver (d), and cardiac (e) indices in hyperlipidemic rats during the treatment.

Six-week food intake, body weight gain, and fat/body ratio of rats are shown in Fig. 1a–c, respectively. The food intake in the HF group was slightly higher than that of the NF and treatment groups during the experimental period, but the difference was not very significant (Figs. 1a and 2a), which indicates that the lipid- and weight-lowering function of CTS and COS is unrelated to appetite suppression, and the minor decrease of food intake in the treatment groups is due to the fact that the rats generated a slight anorectic effect during the long-term HFD intake, and additionally that the very minimum side effects of CTS and COS, including nausea, vomiting, and constipation, also contributed to the reduced food intake (18).

CTS, COSI, and COSIII can lower body weight gains (Fig. 1b, *p*<0.05) and fat/body ratio (Fig. 1c, *p* < 0.05) of rats dose-dependently to the normal range when compared with that in the NF and HF groups.

Figure 1d and e displays the visceral (liver and cardiac) indices (visceral index = visceral mass×100/body mass). The liver index of HF group was clearly higher than that of NF group that is due to the high fat content in HF livers. Treatment experiments revealed that CTS, COSI, and COSIII consistently reduced the liver index in dose-dependent manner (*p* < 0.05). Similarly, cardiac indices in the NF, SV, and all treatment groups were remarkably inferior to that of the HF group (*p* < 0.05), because these foods effectively suppressed the HFD-induced increase in cardiac fat mass and cardiomyocyte hypertrophy. In addition, COSI showed the best decreases of liver (Fig. 2d) and cardiac (Fig. 2e) indices compared with other treatment groups. However, there was no reduction in the kidney index of each treatment group (data not shown).

### Serum lipids

**Fig. 3:**
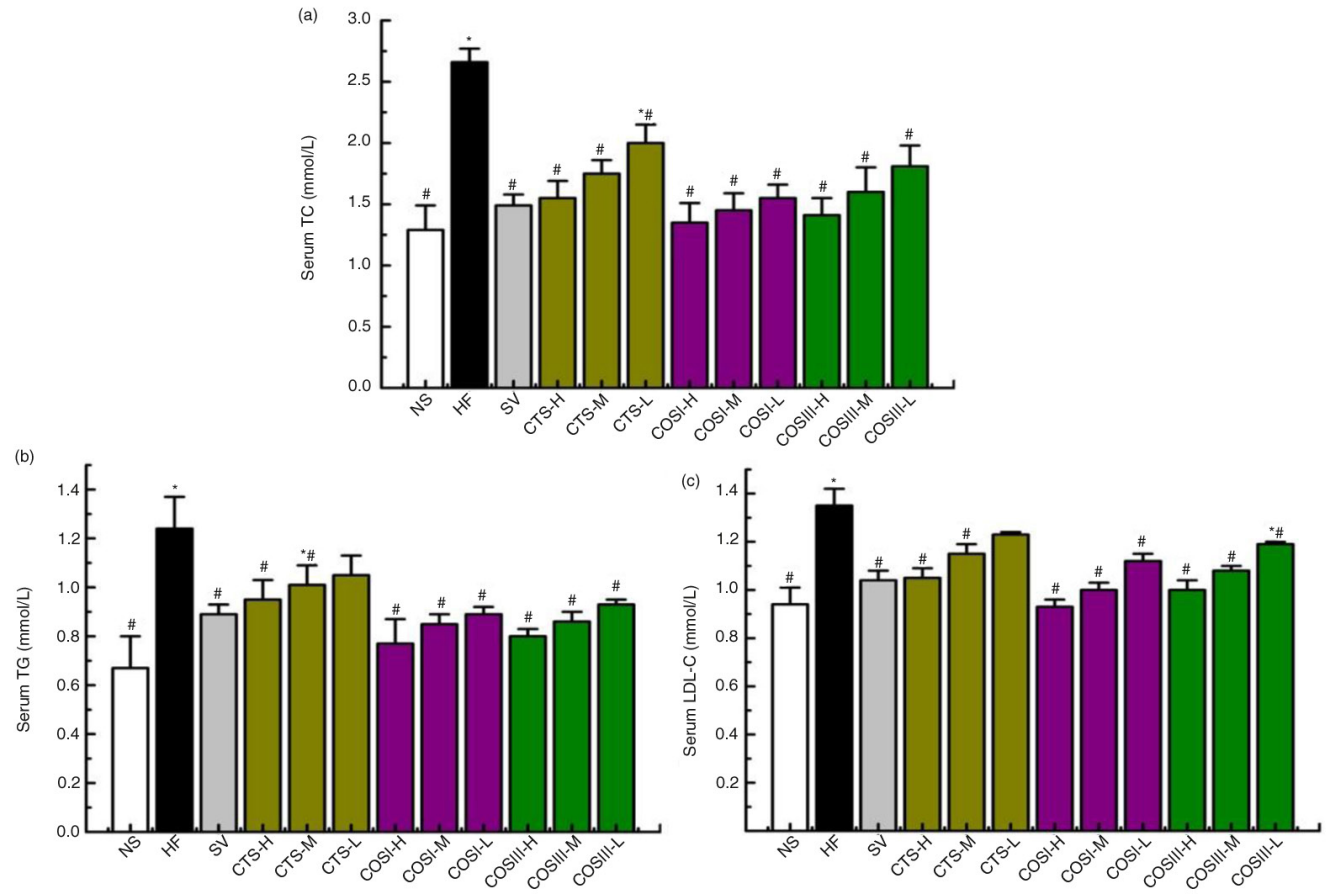
Effects of CTS, COSI, and COSIII on serum TC (a), TG (b), and LDL-C (c) in hyperlipidemic rats during the treatment. The data are expressed as means±SD (*n=*10).

Currently, statins (i.e. simvastatin) are the most commonly used drugs for treating CHC, especially widely used to lower cholesterol and LDL-C levels, and simultaneously simvastatin can effectively increase levels of PPARα in some recent studies, which are the reasons for choosing simvastatin as the positive control material in this study (19). During the experimental period, serum TC, TG, HDL-C, and LDL-C levels were determined after 2 weeks. TC, TG, and LDL-C concentrations increased significantly in all 11 HFD groups (*p<*0.05), which became the selective criterion of HLP rats for the next treatment experiment. Figure 3 displays rat plasma lipids levels after an additional 6 weeks. The HF group displayed significantly higher serum TC, TG, and LDL-C compared with the NF group (*p<*0.05). The high serum TC levels in the CTS, COSI, and COSIII treated groups were significantly reversed in a dose-dependent compared to the HFD group (*p<*0.05), and the therapeutic effects of COSI and COSIII were equivalent to that of SV. Similar changes were also observed for serum TG concentrations, especially for rats fed with COSI and COSIII, which had lower serum TG levels that were similar to the SV group. CTS, COSI, and COSIII also dramatically improved the high plasma LDL-C levels induced by HFD accompanied by not changing the HDL levels.

**Fig. 4:**
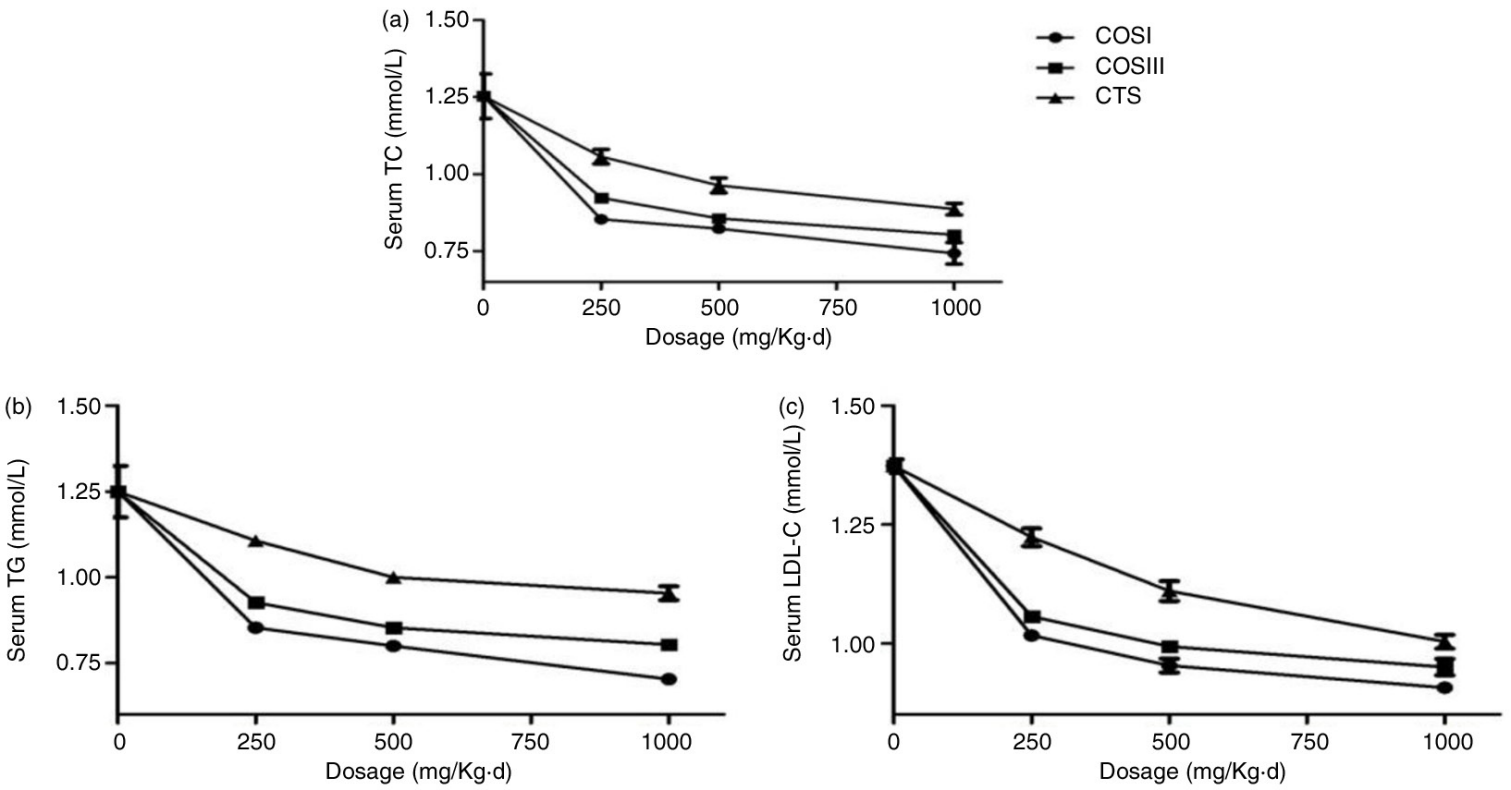
Comparison of COSI, COSIII, and CTS in serum TC (a), TG (b), and LDL-C (c) in hyperlipidemic rats during the treatment.

In a word, this project successfully established a HLP rat model by feeding an optimized HFD, which displayed remarkably increased serum TC, TG, and LDL-C concentrations, as well as relative indices, including liver index, cardiac index, body weight gain, and fat/body ratio, when compared with the normal peers. After the 6-week preventative treatment, these indices above were improved in rats fed with different doses of CTS, COSI, and COSIII, which indicated that CTS and its relative derivatives play the vital role in preventing the invasion of HLP with no effect on the appetite of rats (20). Therein, compared with the SV, CTS, and COSIII groups, the three doses of COSI groups showed the better inhibition of increases in rat body weight gain (Fig. 2b), fat/body ratio (Fig. 2c), especially liver index (Fig. 2d), serum TC, TG, and LDL-C levels (Fig. 4a–c). Given its smaller molecular weight, COSI has the better water-solubility and can be digested and absorbed more easily by the gastrointestinal tract in animals and humans.

### Liver and fecal TC, TG and TBA

It is generally accepted that the positive charge in the structure of CTS and COS can vigorously absorb fat, fatty acids (FA) and bile acids (BA), containing a negative charge, which contributes to lowering serum TC and TG (21). Nonetheless, several studies have described the mechanism by which CTS, COSI, and COSIII reduce lipid levels are related to charge as well as the regulation of liver lipid metabolism (3, 8, 9, 17). To remove excess cholesterol, it must be transported from the peripheral tissues to the liver and intestine, where it is finally excreted in the form of BA via the feces. The metabolic pathway is traditionally considered the RCT or centripetal cholesterol flux pathway (22). In order to determine the effects of the three compounds on the RCT process, we investigated the changes in liver and fecal lipids and BA levels after the administration of these foods.

**Fig. 5:**
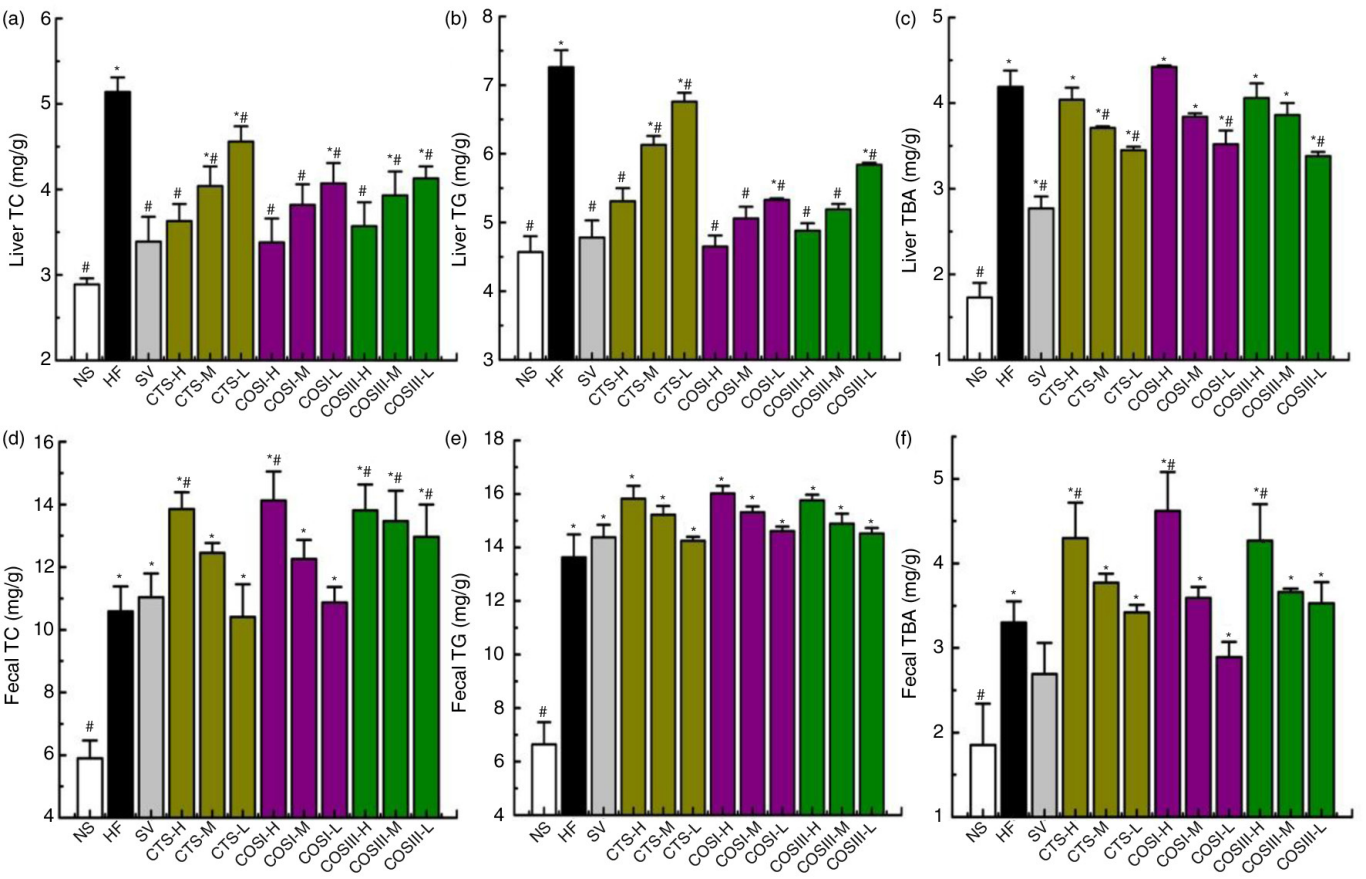
Effects of CTS, COSI, and COSIII on liver TC (a), TG (b), and TBA (c), as well as fecal TC (d), TG (e), and TBA (f) in hyperlipidemic rats during the treatment. The data are expressed as means±SD (*n =* 10).

The liver TC, TG, and TBA levels of tested rats are shown in Fig. 5a–c. TC and TG concentrations in rats fed different doses of CTS, COSI, and COSIII decreased dose-dependently when compared with that in the HF groups, which were consistent with changes in serum TC and TG (*p<*0.05). The results indicated that CTS, COSI, and COSIII can effectually reduce lipid concentration by accelerating the synthesis of TBA in liver and promoting the excretion of cholesterol.

In the following experiments, we determined the concentrations of TC, TG, and TBA in rats’ feces. Figure 5d–f demonstrates that fecal TC, TG, and TBA concentrations of the HF group were higher than that of the NF group (*p* < 0.05). After 6 weeks of treatment, fecal TC, TG, and TBA levels in the treatment groups were elevated in a dose-dependent manner compared with the NF and HF groups (*p* < 0.05).

**Fig. 6:**
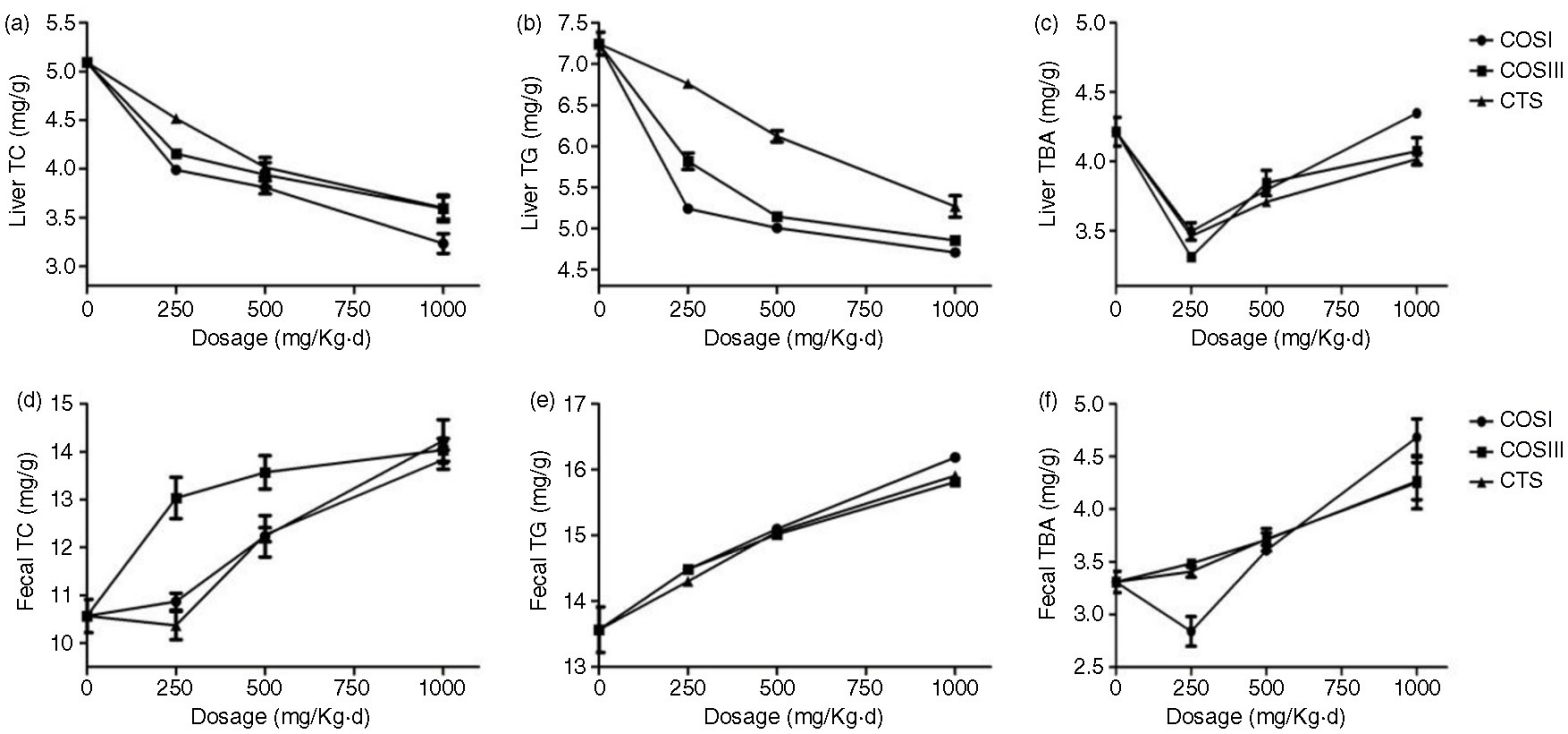
Comparison of COSI, COSIII, and CTS in liver TC (a), TG (b), and TBA (c), as well as fecal TC (d), TG (e), and TBA (f) in hyperlipidemic rats during the treatment.

Therefore, the decreased liver and increased fecal TC, TG, and TBA in the treatment groups indicated that CTS, COSI, and COSIII can promote the RCT process for the excretion of TC, TG, and TBA in hyperlipidemic rats. Specifically, the liver and fecal TBA contents of the HF group were significantly higher than the NF group, because the rise in liver TC and TG contributes to the BA synthesis in the liver, which promotes the excretion of TC and TG in the form of BA via feces. CTS, COSI, and COSIII effectively accelerate the RCT process in HLP rats, as liver BA was markedly higher than the NF group, and BA excretion via feces was greatly increased compared to the HF and NF groups. As we expected that COSI also had a better improvements of lipids and TBA in rat livers (Fig. 6a–c), but the ability to increase fecal lipids and TBA levels was similar with that of CTS and COS (Fig. 6d–f). The reason for that is the three foods share the common excretion pathway, namely feces, although the smaller molecular weight material, COSI, is more apt to improve the RCT process.

### Hepatoprotective effects

**Fig. 7:**
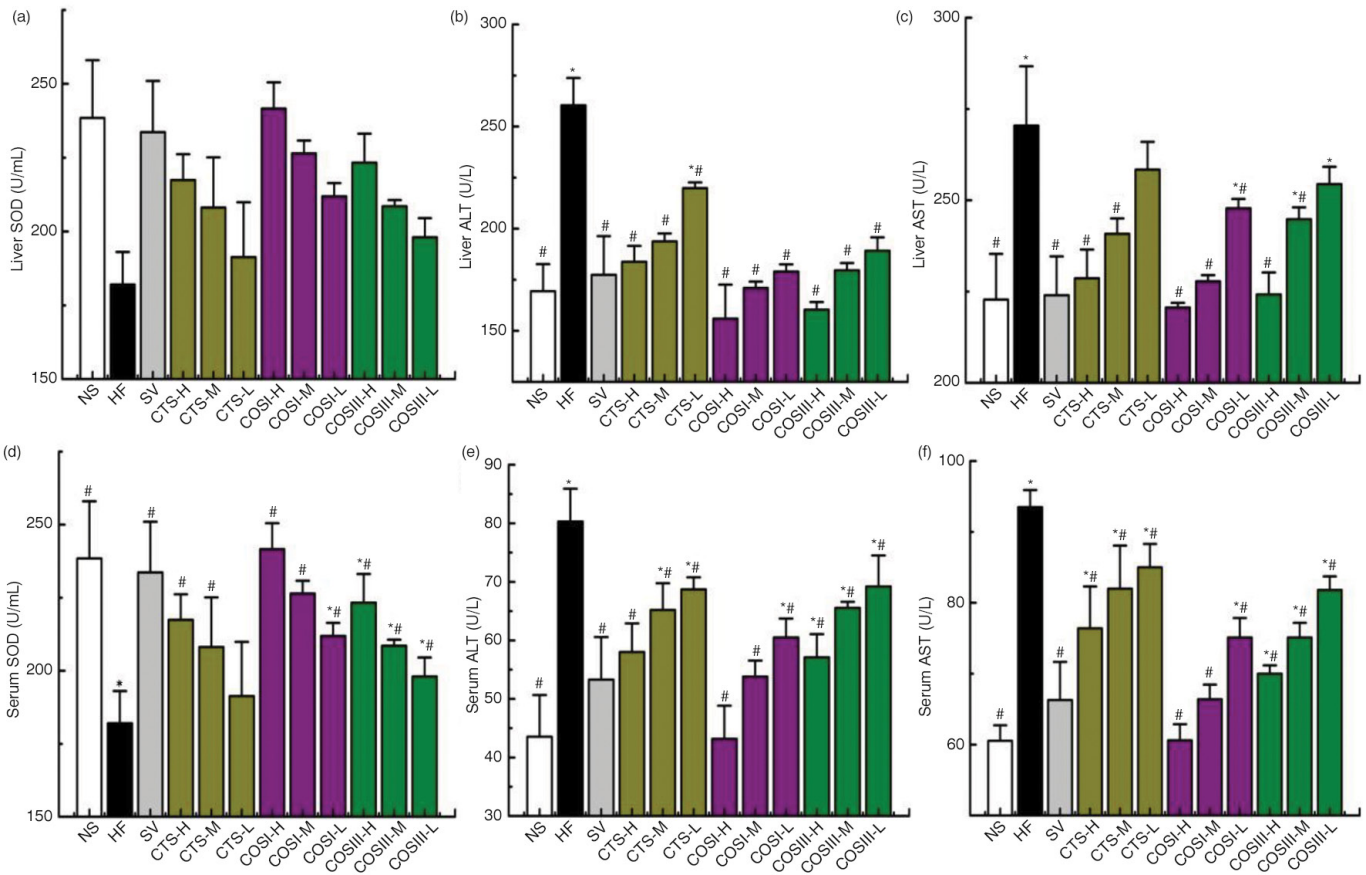
The hepatoprotective effects of CTS, COSI, and COSIII on hyperlipidemic rats. The liver function test, including the determinations of liver (a) and serum SOD (d), serum ALT (e) and AST (f), liver ALT (b) and AST (c), shows that the three compounds can rectify these indices which contribute to normalizing the liver physiological function. The data are expressed as means±SD (*n=*10).

Liver injury, or hepatotoxicity, is the main relative factor of HLP (23). Increased serum ALT and AST damages the structural integrity of the liver because they are located in the cytoplasm and are released into the circulation after hepatocyte damage (24, 25). Elevated liver SOD was accompanied by a decrease in superoxide radicals and further suppression of LDL oxidation, which delays the occurrence and development of AS and HLP (26). The previous research significantly indicated that CTS and its relative derivatives showed antioxidant and hepatoprotective activity *in vivo* and *in vitro* (27, 28). To determine the mechanism underlying the hepatoprotective and antioxidant effects of CTS, COSI, and COSIII, we measured the levels of liver and serum factors regarding liver function and antioxidant properties, including the activities of ALT, AST, and SOD (Fig. 7).

**Fig. 8:**
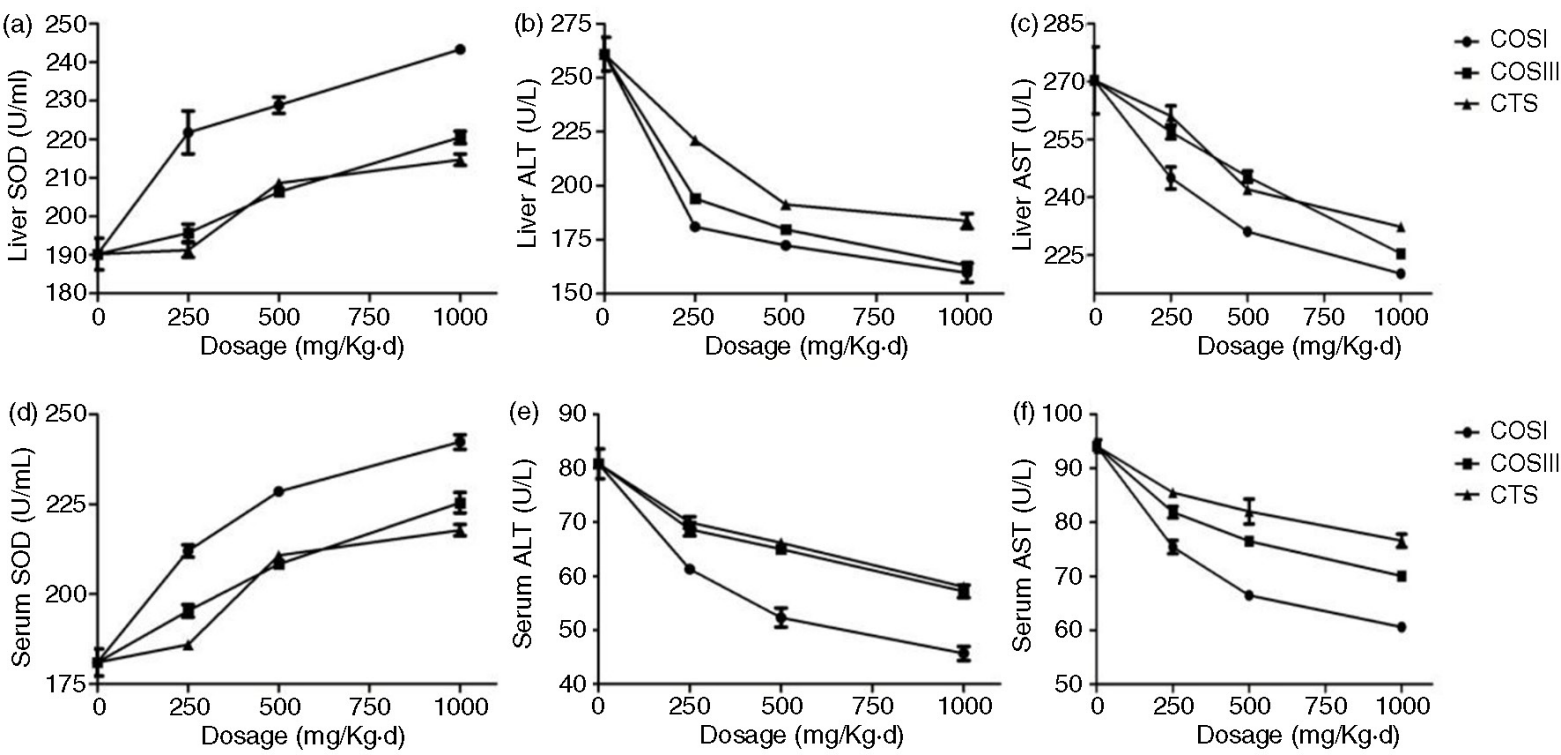
Comparison of COSI, COSIII, and CTS in liver (a) and serum SOD (d), serum ALT (e) and AST (f), liver ALT (b) and AST (c) in hyperlipidemic rats during the treatment.

As shown in Fig. 7b, c, e, and f, rats fed HFD displayed a remarkable increase of ALT and AST in either serum or liver (*p<*0.05) when compared with the NF group. Different doses of COSI, COSIII, and CTS significantly lowered the activities of ALT and AST in serum and liver to the normal range, and particularly the effects of COSI and COSIII in high and middle dose were equivalent to that of SV (*p<*0.05), suggesting that they play an important role in protecting liver function. Meanwhile, liver and serum SOD (Fig. 7a and d) were significantly increased in the SV and treatment groups compared with that in the NF and HF groups, especially in response to different doses of COSI (*p* < 0.05) (Fig. 8), which suggests that the antioxidant activity of CTS, COSI, and COSIII contributes to the hepatoprotective effects described previously.

The expected results clearly demonstrate that CTS, COSI, and COSIII play important roles in preventing liver lipid-oxidation, which contributes to protecting HLP rats’ liver function. Therefore, the hepatoprotective and antioxidant role as well as the cholesterol lowering effects of the three foods could contribute to maintain normal liver function and thus remit the development and/or progression of HLP (29).

### Histopathological responses of liver and adipose tissues

**Fig. 9:**
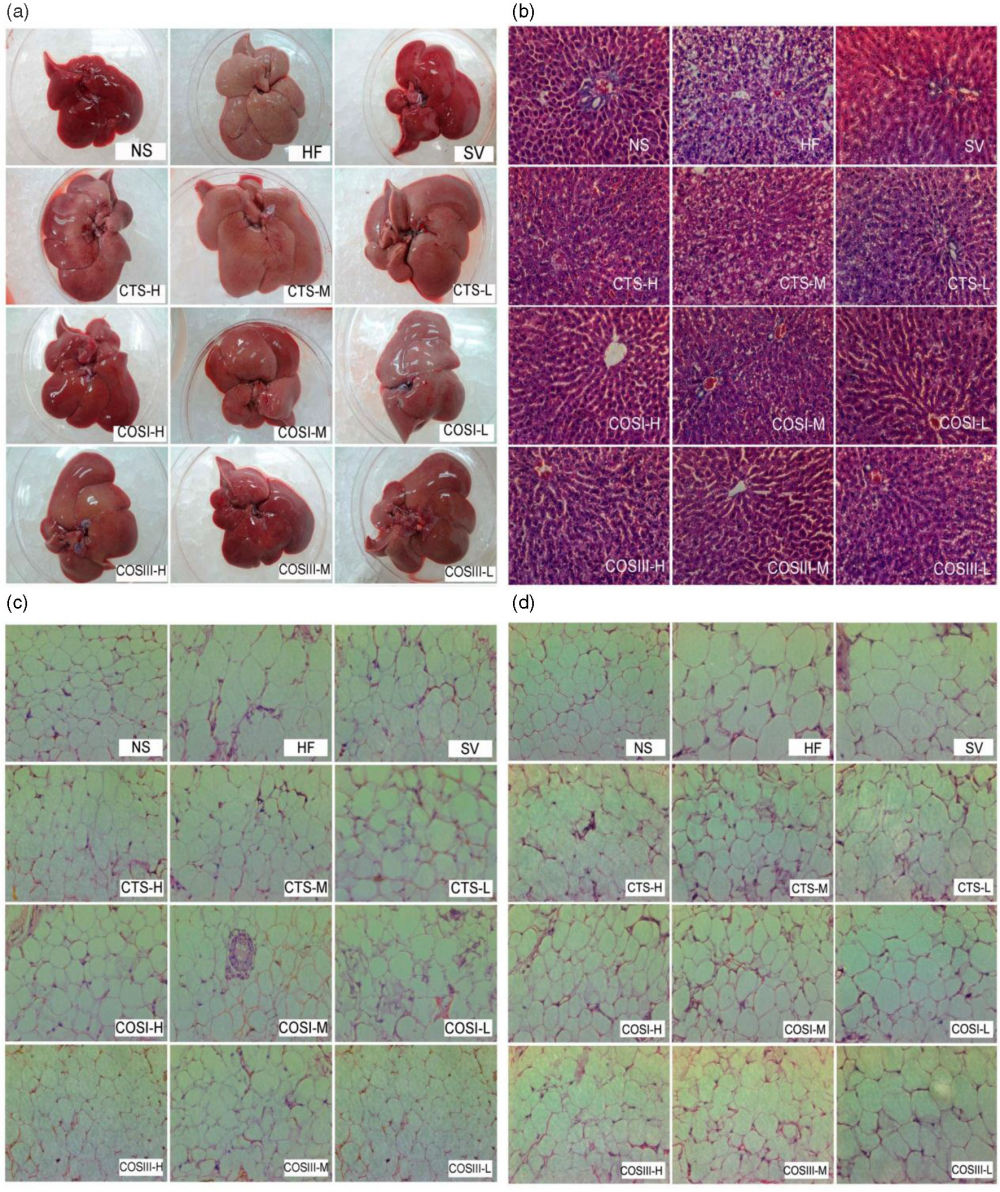
The whole liver (a), slices of liver (b), white subcutaneous, (c) and mesenteric adipose tissues (d) obtained from different groups of SD rats (200×). Tissue sections were stained with hematoxylin and eosin (H&E).

As shown in Fig. 9a, the livers of the NF group were sharp red in color and characterized by being soft, supple, and small in volume, whereas livers in the HF group became dull pale, slightly soft, rich to the touch and swollen, indicating that rats eating the HFD developed serious fatty-liver-like illness. The color of rat livers in the treatment and SV groups was between sharp red and dull pale, specifically in the CTS group. Additionally, the liver morphology of the SV group was similar to that of the NF group, and apart from the three CTS groups, different doses of COSI and COSIII relieved fatty liver to varying degrees.

Tissue sections, including white subcutaneous adipose tissue, mesenteric adipose tissue, and the right lobe of liver tissue, were stained with H&E (Fig. 9b–d). In the NF group, rat livers presented the integrated hepatic lobule and distinct liver sinusoid, and liver cells were neatly distributed around the central vein in a radial pattern. Excess adipose cells were observed in the ampliative central vein surroundings the livers of rats in the HF group. Hepatocytes contained a pale, watery cytoplasm, a cell nucleus that extended to the edge of cytoplasm, and several different shaped lipid droplets in the cytoplasm. Spotty and focal necrosis was observed in hepatic cells from the HF group, and an inflammatory cell infiltrate was observed. Simvastatin effectively reversed the physiological states listed above: the size of liver cells and liver adipose levels returned to normal, and a healthy nucleus was located in the center of sufficient cytoplasm.

The significant decrease of adipocytes and swelling of liver cells in the COSI treatment groups, especially the COSI-H group, suggests that COSI can effectually improve liver lesions. However, the improvements of COSIII and CTS were pale in comparison to COSI. Additionally, CTS, COSI, and COSIII can significantly inhibit the growth of white subcutaneous and mesenteric adipose tissues dose-dependently; this effect was stronger in the COSIII-H group and the three COSI groups.

### Expression of liver PPARα and HL mRNA

Liver lipid metabolism, especially the RCT process, is modulated by various regulatory enzymes, of which HL is the most important. HL can catalyze TG hydrolysis into CM and VLDL-C for tissue use in the form of FFA and monoacylglycerol, and also provide HDL with apolipoproteins and phospholipids for the RCT. Cenarro et al. (30) demonstrated a negative correlation between the development of HLP and liver HL levels. PPARα, a transcription factor for various aspects of liver lipid metabolism, including the uptake, activation, trafficking, β-oxidation, and ketogenesis of FA, and the metabolism of lipoproteins, TG, and TBA can govern the hepatic lipid metabolic processes by increasing the expression of liver HL for the uptake and transportation of FA, which also contributes to the RCT process (31, 32). A number of studies reported that the expression of liver PPARα is down-regulated in hyperlipidemic rats, especially in the hypercholesterolemic rats (33–35). The facts that CTS can reduce the expression and activities of liver HL and PPARα in hyperlipidemic rats compared to controls, and that increased liver HL was invariably correlated with elevated PPARα, indicates the hypolipidemic mechanism of CTS and its derivatives (9).

**Fig. 10:**
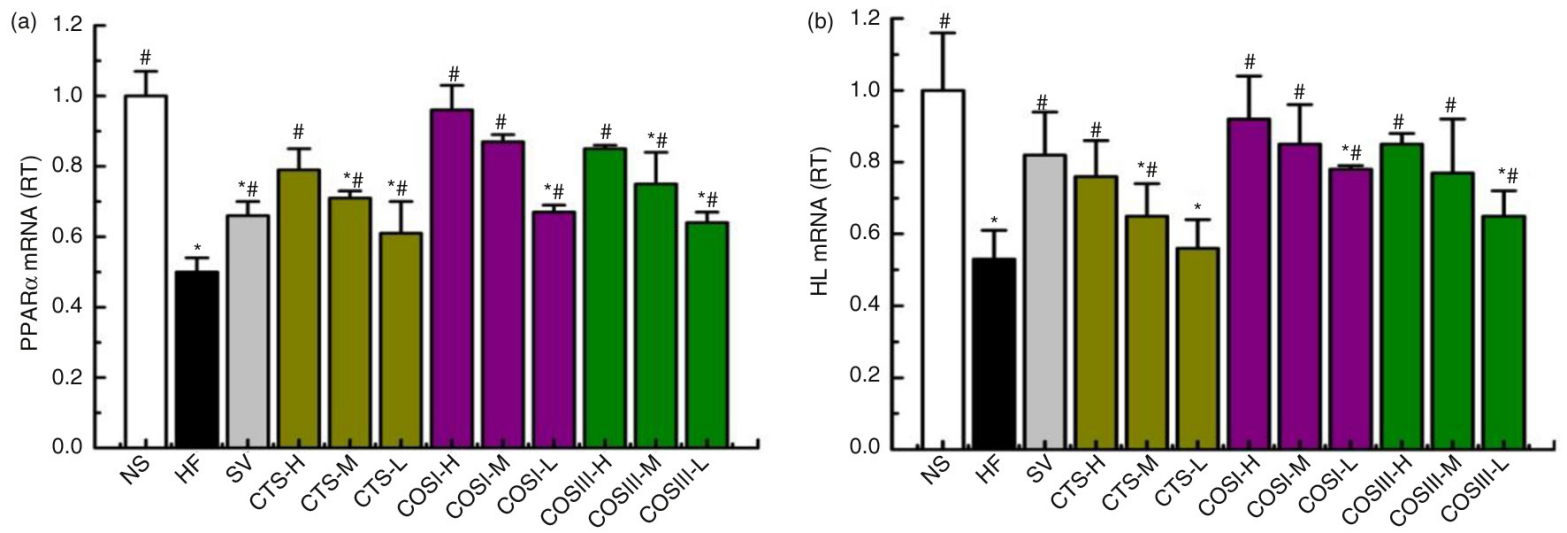
The mRNA expression levels of liver PPARα (a) and HL (b) in the different group. The level of mRNA was detected by Q-PCR. ΔCt is the average value of 10 samples in the formulation (average mRNA expression of experiment groups/average mRNA expression of NF groups) = 2^−ΔΔCt^=^2(−ΔCt control −ΔCt FF)^. If 2^−ΔΔCt^<1, the average mRNA expression of the experiment groups is lower than that in the NF group. If this value is higher than 1, the average mRNA expression of the experiment groups is higher than that in the NF group.

**Fig. 11:**
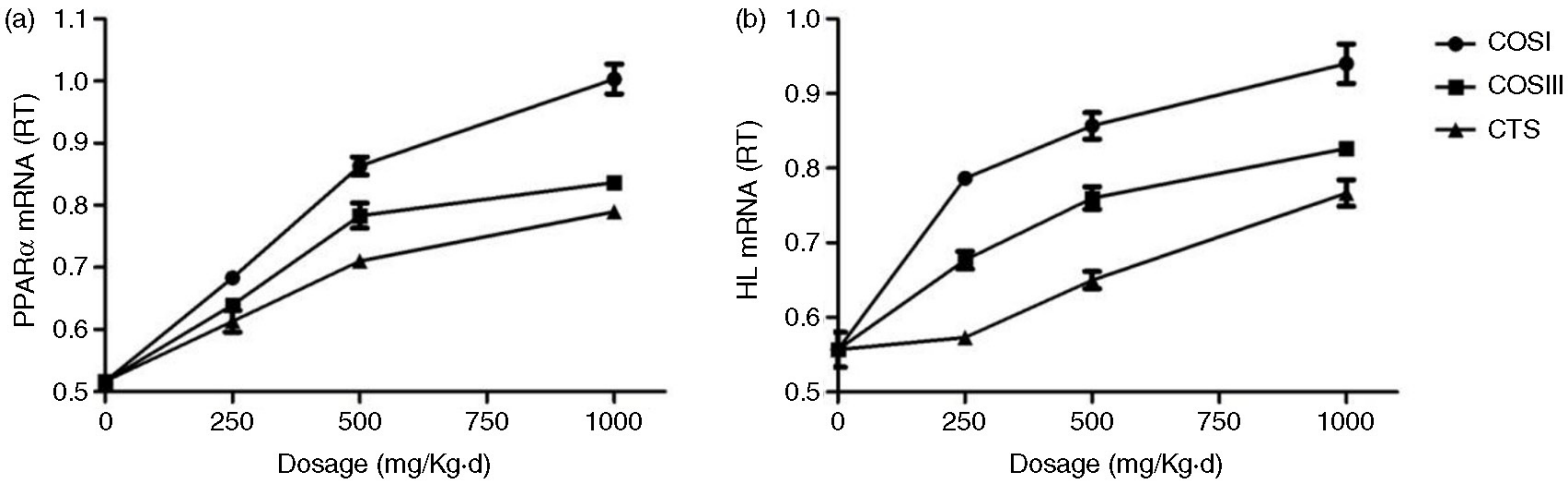
Comparison of COSI, COSIII, and CTS in improvement the mRNA expression levels of liver PPARα (a) and HL (b) in hyperlipidemic rats during the treatment.

In this study, we measured the expressions of rat liver PPARα and HL mRNA using real-time quantitative PCR. Figure 10a demonstrates that PPARα mRNA expression was increased in response to different doses of CTS, COSI, and COSIII, and these differences were statistically significant compared to the HF and NF groups (*p*<0.05). Notably, the effect was stronger in the three COSI groups, especially in COSI-H group (Fig. 11a). As displayed in Fig. 10b, CTS, COSI, and COSIII effectively un-regulated the liver HL mRNA levels in rats fed a HFD, which is similar to liver PPARα mRNA levels changes with the stronger increase in the three COSI groups (Fig. 11b).

In consequence, the increased PPARα and HL are of the dominant factors in controlling the RCT process, which contributes to the improvement of RCT process in the HLP rat model. Simultaneously, we confirmed that simvastatin can also improve the down-regulated PPARα and HL mRNA expressions in liver.

## Conclusions

The obtained results suggested that these three function foods, CTS, COSI, and COSIII, can effectively improve liver lipid metabolism by normalizing the expressions of PPARα and HL, and protect liver from the oxidized trauma by enhancing hepatic function, which could be potentially developed as future functional foods for remedying hyperlipidemia. However, the long-term clinical trials in humans are needed to investigate the hypolipidemic effects of the three function foods and their in-depth mechanisms of lipid-lowering and hepatoprotective effects should be further explored in the future.
